# Y-box binding protein 1 enhances DNA topoisomerase 1 activity and sensitivity to camptothecin via direct interaction

**DOI:** 10.1186/s13046-014-0112-7

**Published:** 2014-12-24

**Authors:** Ying Wu, Ke-yong Wang, Zhi Li, Yun-peng Liu, Hiroto Izumi, Hidetaka Uramoto, Yoshifumi Nakayama, Ken-ichi Ito, Kimitoshi Kohno

**Affiliations:** Department of Medical Oncology, the First Hospital, China Medical University, Shenyang, China; Shared-Use Research Center, School of Medicine, University of Occupational and Environmental Health, Kitakyushu, Fukuoka Japan; Department of Occupational Pneumology, Institute of Industrial Ecological Science, School of Medicine, University of Occupational and Environmental Health, Kitakyushu, Fukuoka Japan; Department of Surgery, School of Medicine, University of Occupational and Environmental Health, Kitakyushu, Fukuoka Japan; Department of Surgery, School of Medicine, Shinshu University, Matsumoto, Nagano Japan; The President Laboratory, University of Occupational and Environmental Health, Kitakyushu, Fukuoka Japan

**Keywords:** Drug resistance, Oxidative stress, Protein interaction domains and motifs, Topoisomerase 1, Y-box binding protein 1

## Abstract

**Background:**

The Y-box binding protein 1 (YB-1) possesses pleiotropic functions through its interactions with various cellular proteins, and its high expression levels make it a potential useful prognostic biomarker for cancer cells. Eukaryotic DNA topoisomerases, such as DNA topoisomerase 1 (TOPO1) and DNA topoisomerase 2 (TOPO2), are the essential DNA metabolism regulators that usually overexpressed in cancer cells, and multiple proteins have been reported to regulate the enzyme activity and the clinical efficacy of their inhibitors. The present study unraveled the interaction of YB-1 with TOPO1, and further investigated the related function and potential mechanisms during the interaction.

**Methods:**

The direct association of TOPO1 with specific domain of YB-1 was explored by co-immunoprecipitation and GST pull-down assays. The interaction function was further clarified by DNA relaxation assays, co-immunoprecipitation and WST-8 assays with in vitro gain- and loss- of function models.

**Results:**

We found that YB-1 interacts directly with TOPO1 (but not with TOPO2) and promotes TOPO1 catalytic activity. Interactions between YB-1 and TOPO1 increased when cancer cells were treated with the TOPO1 inhibitor, camptothecin (CPT), but not with the TOPO2 inhibitor, adriamycin (ADM). Furthermore, we found that the interaction is prevented by pretreatment with the antioxidant agent, N-acetyl cysteine, and that YB-1 downregulation renders cells resistant to CPT.

**Conclusions:**

Our findings suggest that nuclear YB-1 serves as an intracellular promoter of TOPO1 catalytic activity that enhances CPT sensitivity through its direct interaction with TOPO1.

## Background

The Y-box-binding protein-1 (YB-1) plays pleotropic roles in DNA replication, transcription, and repair [1-3]. Previous studies have shown that YB-1 enhances cellular resistance to genotoxic stress through its direct or indirect interactions with several DNA replication and repair proteins [2,4], and that oxidative DNA damage plays an important part in initiation of the repair process [5]. As an important tumor-related protein [1,6-8], YB-1 mediates cellular resistance to anticancer drugs such as cisplatin, adriamycin (ADM), paclitaxel, and etoposide (VP-16) [1,6,7,9-11]. There are many reports that YB-1 is a predictor of clinical outcome in cancer patients [6,12].

DNA topoisomerases are ubiquitous nuclear enzymes that catalyze conformational changes in a double-stranded helix DNA through breakage and rejoining reactions [13]. The activity of these enzymes is essential for various DNA-related processes, such as replication, transcription, chromosome condensation and de-condensation [14]. Thus, DNA topoisomerases are important enzymes during cell proliferation, especially in cancer cells [15]. Topoisomerase-targeting drugs can be used as anticancer agents. Such drugs interfere with the breakage/rejoining activity of these enzymes through the formation of the so-called drug/enzyme/DNA ‘cleavable complex’ [16]. The accumulation of drug-induced cleavable complexes may be cytotoxic [17-19]. An important question, therefore, is how cellular sensitivity to topoisomerase-targeting drugs is controlled [14,20].

Here, we determined that YB-1 binds directly to TOPO1 and functions as an endogenous regulator of TOPO1-dependent DNA relaxation. This suggests that YB-1 is able to interact with greater numbers of TOPO1 molecules during camptothecin (CPT)-induced oxidative-stress and that this process increases cellular sensitivity to this drug.

## Materials and methods

### Cell lines and antibodies

Human prostate cancer cells (PC-3), and gastric cancer cells (HGC-27) and pancreatic cancer cells (PANC-1) were cultured in Eagle's minimal essential medium and RPMI-1640 medium containing 10% fetal bovine serum (Nissui Seiyaku, Tokyo, Japan), respectively. Stable transfectants derived from PC-3 cells were established and maintained as described previously [21]. Antibodies against Lamin B1, TOPO1, TOPO2 and GST were purchased from Santa Cruz Biotechnology (CA, USA). The anti-Flag antibody was from Sigma (MO, USA), and the anti-Thio antibody was from Invitrogen (CA, USA). Anti-YB-1 was generated by immunization of a New Zealand white rabbit with synthetic peptides (C-terminal amino acids 299–313) as described previously [11].

### Small interfering RNAs (siRNAs), WST-8 assay, and Western blot analysis

As described previously [22,23], aliquots of 4 × 10^3^ and 1 × 10^6^ PC-3 cells transfected with specific YB-1 siRNAs (Invitrogen; YB-1 siRNA #1, 5-AAAGCAAGCACUUUAGGUCUUCAGC-3 (sense) and 5-GCUGAAGACCUAAAGUGCUUGCUUU-3 (antisense); and YB-1 siRNA #2, 5-UUUGCUGGUAAUUGCGUGGAGGACC-3 (sense) and 5-GGUCCUCCACGCAAUUACCAGCAAA-3 (antisense)) were used in water-soluble tetrazolium salt (WST-8) assays and western blot analysis, respectively. TetraColor ONE was obtained from Seikagaku Corp.(Tokyo, Japan).The following antibodies and dilutions were used: a 1:1,000 dilution of anti-TOPO1, anti-TOPO2, anti-Lamin B1, anti-Thio, and a 1:5,000 dilution of anti-YB-1 and anti-Flag. Column diagrams beside each western blot illustrated data as the mean (± SD) ratio of TOPO1 signals over the affinity-precipitated flag-YB-1 signals.

### Plasmids, recombinant proteins, and chemical reagents

The *Escherichia coli* plasmid expression constructs containing full-length GST-YB-1 cDNA, three GST-YB-1 deletion mutants (GST-YB-1Δ1, Δ2, and Δ3), and the mammalian plasmid expression construct, pcDNA3-Flag-YB-1, were described previously [24]. Full-length TOPO1 cDNA was kindly provided by Dr. Toshio Ando. The cDNA fragment was purified and cloned into pThioHis (Invitrogen) for expression in bacterial cells. Glutathione *S*-transferase (GST) and ThioHis fusion proteins were induced by 1 mM isopropyl-beta-D-thiogalactopyranoside (IPTG; Sigma), purified with glutathione beads (GE Healthcare), and eluted with glutathione elute buffer (50 mM Tris–HCl, 10 mM reduced glutathione, pH 8.0). Human TOPO1 was purchased from TopoGEN, Inc. (Ohio, USA). CPT was supplied by Calbiochem-Novabiochem Corp. (CA, USA). N-acetyl-L-cysteine (NAC) was from Sigma. ADM was a kind gift from Kyowa Hakko Kogyo Co., Ltd. (Tokyo, Japan).

### Co-immunoprecipitation and GST pull-down assay

Co-immunoprecipitation was performed as described previously [21]. For the interactions between endogenous YB-1 and TOPO1, the cytosolic or nuclear extracts (500 μg) from transfection-minus cells were treated with nuclease and incubated for 4 h at 4°C with 2 μg of goat IgG, anti-TOPO1, or anti-TOPO2 antibody. Protein A-Sepharose beads (GE Healthcare) were added and incubated for 2 h at 4°C with the extracts. To detect drug induced binding of YB-1 and TOPO1, stable PC-3-transfectants expressing Flag-YB-1, were cultured in 100-mm tissue culture plates in the presence or absence of the chemical reagents indicated. Nuclear extracts (500 μg) were prepared and incubated for 4 h at 4°C with 10 μl of anti-Flag M2 magnetic beads (Sigma). Immunoprecipitated samples were washed thrice with lysis buffer, and together with pre-immunoprecipitated samples (input, 50 μg) were subjected to SDS-PAGE and Coomassie blue staining, or western blot analysis. Expression of ThioHis-TOPO1, GST-YB-1, and serial deletion mutants of GST-YB-1 in bacteria and the GST pull-down assays were carried out using glutathione-sepharose 4B (GE Healthcare), as described in our previous reports [4,25,26] and the current figure legends.

### DNA relaxation assays

TOPO1 activity was measured by the relaxation of supercoiled pGEM-T easy plasmid (Promega, Madison, WI, USA). The assay mixture consisted of 1 ng of TOPO1, 100 mM Tris–HCl (pH 7.9), 10 mM EDTA, 1.5 M NaCl, 1 mM spermidine, 50% glycerol, and 1% BSA. GST or GST fusion proteins (40 or 400 ng amounts) were premixed with the assay mixture and incubated at room temperature for 30 min. The reaction was initiated by the addition of 0.25 μg of pGEM-T easy and allowed to proceed at 37°C for 30 min. Reaction products were run on 0.8% agarose gels at 80 V for 40 min in TBE buffer (89 mM Tris, 89 mM boric acid, and 2 mM EDTA, pH 8.0). Gels were stained with ethidium bromide (0.5 μg/ml) for 20 min. Bands were visualized by illumination from below with short-wave length UV light and were photographed.

### Statistical analysis

All experiments were performed at least three times to verify the reproducibility of the findings. An unpaired t-test was used for statistical analysis. p <0.05 was considered statistically significant.

## Results

### YB-1 binds to TOPO1 via its cold shock and C terminal domains

**Figure 1:**
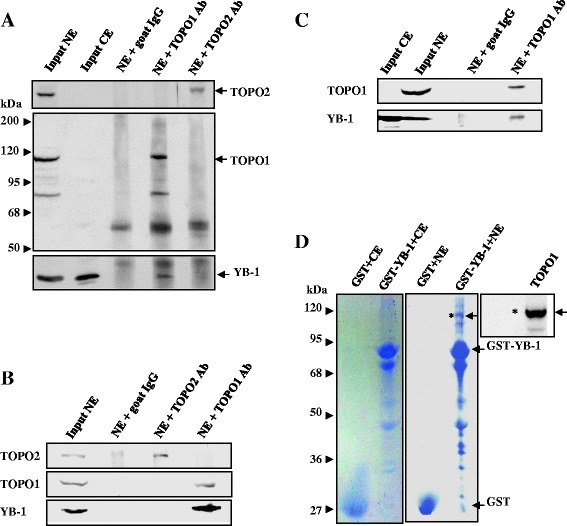
**YB-1 binds to TOPO1. A**. Endogenous YB-1 binds to endogenous TOPO1 in human gastric cancer cells. Nuclear extracts (NE) from HGC-27 cells were subjected to immunoprecipitation with anti-TOPO1 or anti-TOPO2 antibodies, or a goat-IgG antibody control. The immunoprecipitates and the input from NE and cytosolic extracts (CE) were subjected to western blot analysis for TOPO2 (upper panel), TOPO1 (middle panel) and YB-1 (lower panel). **B**. Endogenous YB-1 binds to endogenous TOPO1 in human pancreatic cancer cells. Nuclear extracts (NE) from PANC-1 cells was subjected to immunoprecipitation with a TOPO1 antibody, TOPO2 antibody, or a goat-IgG antibody control. The immunoprecipitates and input were subjected to western blot analysis for TOPO2 (upper panel), TOPO1 (middle panel) and YB-1 (lower panel). **C**. Endogenous YB-1 binds to endogenous TOPO1 in human prostate cancer cells. Nuclear extracts (NE) from PC-3 cells was subjected to immunoprecipitation with a TOPO1 antibody, or a goat-IgG antibody control. The immunoprecipitates and the input from NE and cytosolic extracts (CE) were subjected to western blot analysis for TOPO1 (upper panel), and YB-1 (lower panel). **D**. TOPO1 in cell extracts binds to GST-YB-1 protein. Purified GST or GST-YB-1 protein was incubated, respectively, with 500 μg of cytosolic extracts (CE) and nuclear extracts (NE) from PC-3 cells for 4 h at 4°C. Proteins pulled down with the bait (which was immobilized on glutathione beads) were separated by SDS-PAGE. And then two parallel gels were subjected to western blot and Coomassie blue staining, respectively. Proteins transferred to the membrane were probed with an anti-TOPO1 antibody. The band labeled by an asterisk was identified as TOPO1.

**Figure 2:**
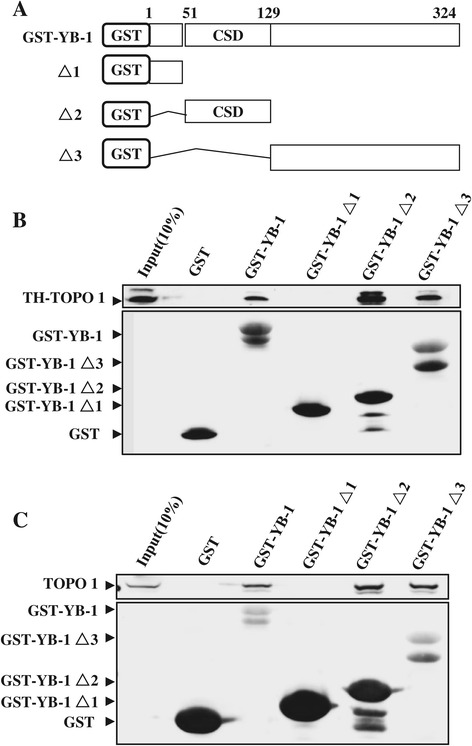
**Identification of the TOPO1 binding domain in YB-1. A**. Schematic illustration of the GST-YB-1 deletion mutants (Ise et al., 1999 [26]). CSD indicates the cold shock domain. Amino acid residues are numbered. **B**. Interaction of GST-YB-1 deletion mutants with ThioHis-TOPO1. Approximately 500 μg of each GST fusion protein was immobilized on 15 μl of glutathione-Sepharose 4B, and the resin was incubated with 500 μg of ThioHis-TOPO1. Resin bound proteins were examined by western blotting using anti-Thio (upper panel) or anti-GST (lower panel) antibodies. **C**. Interaction of GST-YB-1 deletion mutants with TOPO1 in nuclear lysis solutions of PC-3 cells. Approximately 500 μg of each GST fusion protein was immobilized on 15 μl of glutathione-Sepharose 4B, and the resin was incubated with 500 μg of PC-3 nuclear lysis. Resin bound proteins were western blotted and the membrane probed using TOPO1 (upper panel) or anti-GST (lower panel) antibodies.

To determine whether TOPO1 interacted with YB-1 in mammalian cells, we performed co-immunoprecipitation experiment. In human gastric cancer HGC-27 (Figure 1A) and pancreatic cancer PANC-1 cells (Figure 1B), TOPO2 and TOPO1 were observed in the nuclear extracts and immunoprecipitates with the TOPO2 and TOPO1 antibody, respectively (upper and middle panel). In the co-immunoprecipitation assays (lower panel), YB-1 was observed in both nuclear and cytosolic extracts. YB-1 was also found to interact with TOPO1, but not TOPO2 or goat IgG. Endogenous YB-1-TOPO1 interaction was further confirmed in human prostate cancer PC-3 cells (Figure 1C), but not in human lung cancer A549 and cervical cancer HeLa cells (data not shown). To establish whether this interaction was also observed *in vitro*, we performed GST pull-down assays, respectively, using cytosolic extracts and nuclear extracts from PC-3 cells, with either GST-YB-1 or GST protein as the bait (Figure 1D). As anticipated, GST-YB-1, but not GST, bound TOPO1. To determine whether YB-1 interacted with TOPO1 directly, and to identify the TOPO1 binding region in YB-1, we performed pull-down assays using a recombinant ThioHis-TOPO1 fusion protein and GST fusion proteins containing either full-length YB-1 or its mutant derivatives, GST-YB-1 Δ1–Δ3 (Figure 2A). ThioHis-TOPO1 bound to GST-YB-1, GST-YB-1 Δ2, Δ3, but not to GST-YB-1 Δ1 (Figure 2B). With the nuclease-treated PC-3 nuclear extracts, we further confirmed the direct association of YB-1 and TOPO1 in the cells. As seen in Figure 2C, GST-YB-1, GST-YB-1 Δ2, Δ3, but not GST or GST-YB-1 Δ1, bound TOPO1. These findings indicate that YB-1 binds directly to TOPO1 in the nucleus of human cancer cells, and the binding sites were specified as the cold shock domain (CSD) and the C-terminal region of YB-1.

### In-vitro and in-vivo DNA relaxation assays reveal that YB-1 aggregates TOPO1 thereby enhancing DNA relaxation

**Figure 3:**
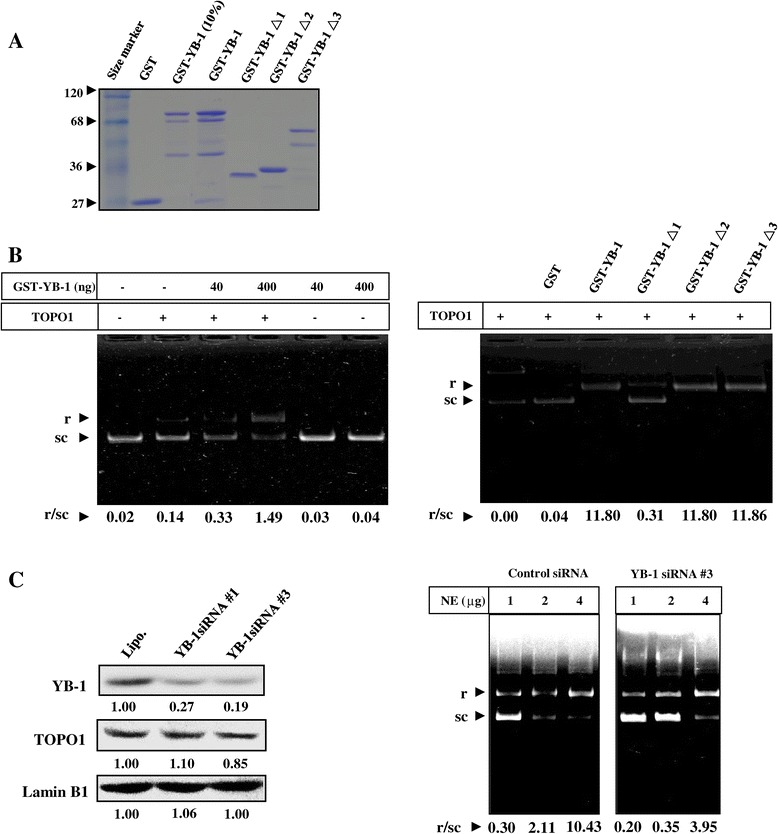
**YB-1 promotes TOPO1 activity in DNA relaxation assays. A**. Purification of recombinant proteins for DNA relaxation assays. Full-length YB-1 or YB-1 deletion mutants were expressed in bacterial cells, purified with 15 μl of glutathione-Sepharose 4B, and subjected to SDS-PAGE and Coomassie blue staining. **B**. Recombinant YB-1 promotes relaxation of supercoiled DNA. pGEM-T easy supercoiled DNA (0.25 μg) was incubated with TOPO1 (1 ng), GST, GST-YB-1 (40 or 400 ng) protein, or a combination of TOPO1 and GST-YB-1 for 30 minutes at 37°C (left panel). To test which part of YB-1 contained the TOPO1-binding domain, pGEM-T easy supercoiled DNA (0.25 μg) was incubated with TOPO1 (1 ng) alone, and TOPO1 (1 ng) with either GST, GST-YB-1, GST-YB-1 Δ1, GST-YB-1 Δ2, GST-YB-1 Δ3, or GST (400 ng) protein, for 30 minutes at 37°C (right panel). The DNA was resolved on agarose gels (without ethidium bromide), and stained thereafter with ethidium bromide. The supercoiled (sc) and relaxed (r) DNA bands are shown. **C**. Endogenous YB-1 knockdown with siRNA reduces TOPO1 DNA relaxation activity. PC-3 cells were transiently transfected with human YB-1 siRNA or control siRNA, and nuclear extracts (50 μg) were subjected to SDS–PAGE and western blotting. Transferred proteins were probed with anti-YB-1 and anti-TOPO1 antibodies, using anti-laminB1 antibodies as a loading control for nuclear protein (left panel). Forty-eight hours after the aforementioned transfection, various amounts of PC-3 nuclear extracts (NE) (1 μg to 4 μg of NE prepared by dilution in phosphate-buffered saline) were incubated with pGEM-T easy supercoiled DNA (0.25 μg) for 30 minutes at 37°C. The supercoiled and relaxed DNA bands are shown in the right panel.

To explore whether the YB-1-TOPO1 interaction modulates the primary function of TOPO1, DNA relaxation assays were performed. The purified recombinant proteins used for DNA relaxation assays are shown in Figure 3A. As seen in Figure 3B, TOPO1 caused relaxation of the supercoiled DNA, whereas GST-YB-1 enhanced the TOPO1-induced DNA relaxation (left panel). With TOPO1, increasing the amount of GST-YB-1 resulted in enhanced DNA relaxation. In contrast, GST and GST-YB-1 alone had no DNA relaxation activity. Furthermore, both the CSD and C-terminal of YB-1 exhibited similar activity profiles to the full-length YB-1 in enhancing TOPO1 induced DNA relaxation (right panel), thereby implying YB-1-TOPO1 binding may be essential for the promotion of TOPO1 activity. To determine whether the YB-1-TOPO1 interaction in cancer cells influences TOPO1-driven DNA relaxation, endogenous YB-1 was knocked down in PC-3 cells with YB-1 siRNA and nuclear extracts were analyzed using *in-vitro* DNA relaxation assays. As seen in Figure 3C, knockdown of YB-1 expression had no impact on TOPO1 expression, but resulted in decreased TOPO1 activity in the 2 μg to 4 μg nuclear extracts of the PC-3 cells. These results therefore demonstrate the enhancement of a functional component of TOPO1 activity in the cells by endogenous YB-1.

### YB-1-TOPO1 association responses to DNA-damaging agents

**Figure 4:**
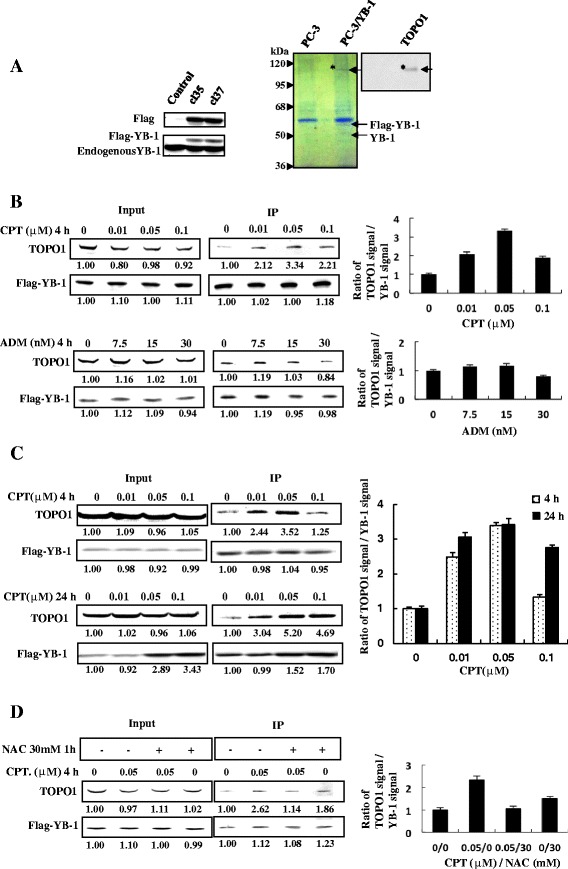
**Chemical treatment promotes binding of TOPO1 and YB-1 through oxidative stress. A**. Identification of the YB-1- TOPO1 interaction in Flag-YB-1 stably-transfected PC-3 cells. Whole-cell lysates (500 μg) prepared from two clones (cl35 and cl37) of PC-3 cells transfected with the pcDNA3-Flag-YB-1 expression plasmid were subjected to SDS–PAGE and western blotting. Transferred proteins were probed with anti-Flag (upper panel) or anti-YB-1 (lower panel) antibodies. Both Flag-YB-1 and endogenous YB-1 are shown (left panel). Nuclear extracts (500 μg) of control cells or cl37 PC-3 cells were immunoprecipitated with the anti-Flag (M2) antibody. The resulting immunocomplexes were separated by SDS-PAGE. And then two parallel gels were subjected to western blot and Coomassie blue staining, respectively. Transferred proteins were probed with the anti-TOPO1 antibody. The band labeled with an asterisk was identified as TOPO1. The Flag-YB-1 protein band is indicated by an arrow. **B**. Chemical treatment increases YB-1-TOPO1 complex. Cl37 Flag-YB-1 stably-transfected PC-3 cells were treated for 4 h with the concentrations of CPT or ADM indicated. Nuclear extracts were immunoprecipitated with the anti- Flag (M2) antibody, and the resulting immunocomplexes were subjected to western blot analysis with anti-Flag and anti-TOPO1 antibodies. **C**. Longer CPT treatment increased YB-1-TOPO1 interaction and YB-1 expression. Cl37 Flag-YB-1 stably-transfected PC-3 cells were treated with the indicated concentrations of CPT for 4 h and 24 h respectively. Nuclear extracts were immunoprecipitated with the anti-Flag (M2) antibody, and the resulting immunocomplexes were subjected to western blot analysis with anti-Flag and anti-TOPO1 antibodies. **D**. Pretreatment with NAC prevented the chemically augmented YB-1-TOPO1 interaction. Cl37 of Flag-YB-1 stably-transfected PC-3 cells were untreated or preincubated with NAC (30 mM, 1 h) before addition of CPT. Nuclear extracts were immunoprecipitated with the anti-Flag (M2) antibody, and the resulting immunocomplexes were subjected to western blot analysis with anti-Flag and anti-TOPO1 antibodies.

To determine the physiologically relevance of YB-1-TOPO1 association in cells, further co-immunoprecipitation was performed with a stable PC-3 cell line expressing a Flag-YB-1 construct. Of the two clones generated, clone 37 (cl37) with slightly higher expression of Flag-YB-1 (left panel, Figure 4A) was used for further experiments, and the immunoprecipitation results showed that the Flag-YB-1 precipitate contained TOPO1 protein (right panel). As seen in Figure 4B, YB-1-TOPO1 complex formation increased respectively by 334% and 221% after 4 h treatment with a 0.05 and 0.1 μM concentration CPT, while no significant increase was observed in YB-1 expression. There was no significant increase in the quantity of the YB-1-TOPO1 complex after ADM treatment compared with untreated PC-3 cells. Higher concentrations of these drugs were toxic to the cells (data not shown). Comparing with 4 h, 24 h incubation of cells with CPT resulted in more YB-1-TOPO1 complex formation and an increase in YB-1 expression. At 24 h treatment, CPT increased YB-1-TOPO1 complex formation and YB-1 expression by 520% and 152%, and by 469% and 170% at the concentration of 0.05 and 0.1 μM, respectively. However, the relative ratio of TOPO1 over YB-1 signals demonstrated no significant difference between 4 h and 24 h incubation when CPT was applied at the concentration not more than 0.05 μM (Figure 4C). Furthermore, pretreatment of PC-3 cells with N-acetyl-cysteine (NAC), which can inhibit reactive oxygen species (ROS) generation [27,28], prevented the CPT-induced increase in YB-1-TOPO1 complexes (Figure 4D).

### Depleting endogenous YB-1 does not affect TOPO1 expression, but induces CPT resistance

**Figure 5:**
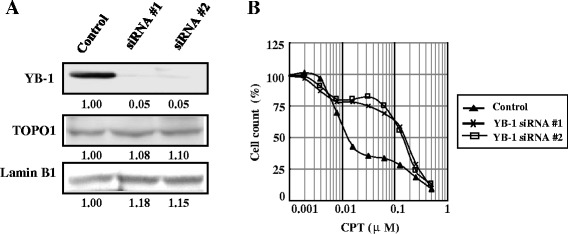
**Impact of depleting endogenous YB-1 on PC-3 survival after CPT treatment. A**. Downregulation of YB-1 expression by two different YB-1 siRNAs. Control siRNA (50 pmol) or YB-1 siRNA were transfected into PC-3 cells, and whole-cell lysates (50 μg) were subjected to SDS–PAGE. Transferred proteins were probed with anti-YB-1 and anti-LaminB1 antibodies. **B**. Knockdown of YB-1 renders cells resistant to CPT. PC-3 cells were treated with 50 pmol of YB-1 siRNA or 50 pmol of control siRNA for 24 h, and then exposed to various concentrations of CPT for 48 h. Cells were stained with TetraColor ONE and their absorbance was measured at 450 nm. All values were the mean of least three independent experiments.

We investigated the effect of knocking down YB-1 in PC-3 cells under CPT treatment. Figure 5A shows that YB-1 expression levels dropped by approximately 90% compared with the control siRNA, and that TOPO1 expression remained constant in the samples. Interestingly, YB-1 depletion in PC-3 cells decreased the cellular sensitivity to CPT (Figure 5B).

## Discussion

YB-1 plays an important role in genome stability and cellular stress responses through interaction with multiple proteins involved in DNA transcription, translation, and repair [1,4,5,29]. Of the three domains YB-1 comprises [4,30], the N-terminal domain (amino acid (aa) 1–50) is critical for transcriptional regulation [31], the central cold shock domain (CSD: aa 51–129) has been recently revealed as the binding site for DNA repair proteins [2,29], and the carboxy-tail domain (aa 130-C-terminus) is thought to interact with other cellular proteins or nucleic acids [4,26,32]. Here YB-1 was shown to associate with the cellular essential DNA regulator TOPO1 through its CSD and C-tail domain (Figures 1 and 2). Previously, this type of joint binding was seen between YB-1 and several transcription factors [1]. Our result not only gives a meaningful supplement to the protein interaction spectrum of YB-1, but also provides a significant entry point for further exploration of both YB-1 and TOPO1.

In an *in-vitro* DNA relaxation assay, we found that YB-1 promoted the DNA relaxation activity of TOPO1 (Figure 3). Previous studies showed that YB-1 binding to DNA significantly decreased the melting temperature of the double helix and resulted in the generation of nuclease-sensitive regions in the DNA [1]. However, YB-1 itself was unable to separate DNA duplex strands larger than 36 bp, although it also exhibited weak nuclease activity [2,24]. As is known, relaxation of DNA supercoiling is a key function of TOPO1, which separates the DNA duplex into single strands for replication, transcription, and repair [13]. TOPO1 activity is affected by variety of cellular proteins through direct associations [33,34]. YB-1 interacts with DNA when associated with other proteins and involves in almost all DNA dependent processes through regulating the activity of its binding proteins [1-4,26]. Therefore, YB-1 might bind DNA as a complex with TOPO1 and is involved in DNA dependent processes by providing an appropriate environment for TOPO1 to relax the DNA duplex.

Both YB-1 and TOPO1 are considered important for cellular stress responses and genomic stability, and can recognize DNA lesions [1,13,33]. Therefore, the interaction between YB-1 and TOPO1 could have important implications for cancer responses to chemotherapy. Currently, CPT (Figure 4B) increased YB-1-TOPO1 complex formation in PC-3 cells, and the increase of YB-1-TOPO-1 complex was almost equal between 4 and 24 h incubation when CPT was applied at a relative lower concentration (Figure 4C). Moreover, CPT-induced YB-1-TOPO1 complex formation was prevented by antioxidant NAC (Figure 4D). However, the expression levels of YB-1 and TOPO1 showed no obvious alterations after 4 h drug treatment of the cells (Figure 4B-4D). This suggests that YB-1-TOPO1 interaction is affected at least partly by drug-induced oxidative stress.

Notably, YB-1-TOPO1 association demonstrated no significant different in PC-3 cells treated by ADM (Figure 4B). Different with CPT, which targets TOPO1 and induces DNA damage in cancer [28,35,36] by increasing cellular ROS [37-39], ADM targets TOPO2 and its cytotoxicity towards cancer cells does not originate from oxidative stress, as demonstrated by the fact that antioxidant treatment can not decrease ADM-induced apoptosis in cancer cells [40]. Furthermore, particular oxidative DNA damage promotes TOPO1 DNA binding and cleavable complexes formation [13,41-43], or induces multiple interactions between YB-1 and various DNA repair proteins [2,4,5]. Therefore, YB-1-TOPO1 interaction might react to drug-specific oxidative cytotoxicity in cancer cells, which warrants further in-depth researches.

As for the dose-dependent YB-1-TOPO1 interaction perturbations in cells treated by CPT, it is an intriguing phenomenon deserves further discussion. The toxicity of CPT on cells has been reported to be dose-dependent [44]. As demonstrated by morphological and molecular changes detected as early as 3 h post exposure, lower (<0.1 μM) concentrations led to reversible cessation of chromosome synthesis, while higher (≥0.1 μM) ones caused irreversible cell cycle arrest followed by apoptosis [44,45]. Moreover, the different cell fates relied on the degree of DNA damage. Under distinct concentration of CPT treatment, the reversible cellular changes were attributed to gene repair induced by mild DNA damage, whereas permanent changes related with apoptosis by extensive DNA damage [44,46]. Currently, YB-1-TOPO1 interaction climbed up in cells with 0.01 μM- and 0.05 μM- CPT treatment, and then declined when the concentration of CPT reached 0.1 μM (Figure 4C). Considering the important role of YB-1 for DNA repair [1], YB-1 might associate with TOPO1 to mediate transient suspension of chromatin synthesis for DNA repair processes, whereas break away from TOPO1ccs to make way for apoptosis once the irreversible cell damage is initiated, and 0.05-0.1 μM might be the turning point in our current study. Besides the dose-dependent YB-1-TOPO1 interaction perturbations, the deduction could be well supported by our further assays where the viability differences by YB-1 depletion in CPT-treated cells gradually increased and then reduced under the concentration ladder of CPT between 0.01 and 0.1 μM (Figure 5B). Further studies are warranted to declare the underlying mechanisms.

CPT compounds specifically inhibit TOPO1, and their efficacy can be affected by TOPO1 expression level and enzyme activity [13,33,47]. Various factors have been found to regulate cellular sensitivity to these compounds and affect their clinical application [13,14,47,48]. We currently found that decreased YB-1 reduced the sensitivity of PC-3 cells to CPT (Figures 5B) while having no effect on TOPO1 expression (Figures 3C and 5A). This result could be explained as follows. First, resistance to CPT seems to be partly caused by low TOPO1 activity [13,14,48], a finding that is consistent with our own YB-1 depletion experiments. Second, CPT induces cytotoxicity by specifically trapping and stabilizing drug-induced cleavable complexes [13,48]. As YB-1 was presently shown to bind TOPO1 directly and promote its activity, knockout YB-1 might have created conditions that impaired the formation or the normal structure of the cleavable complex, thereby decreasing cellular sensitivity to CPT. Third, YB-1 silencing specifically reduced S-phase contents of cells [49], which are most sensitive to CPT treatment [50], therefore results in decreased CPT toxicity. Although YB-1 was commonly regarded to mediate cellular resistance to multiple drugs, such as cisplatin, by up-regulating some ABC transporters and DNA repair proteins, no evidence by far indicates the involvement of YB-1 on the regulation of important CPT inactivators, such as ABCG2, Poly (ADP-ribose) polymerase 1 (PARP-1) and tyrosyl-DNA phosphodiesterase1 (TDP1). Thus, it is reasonable that YB-1 increases cellular sensitivity, but not resistance to CPT. Given that CPT is usually combined with cisplatin, and some other drugs for anticancer treatment [51,52], the opposite effect of YB-1 on cellular sensitivity to CPT and other anticancer drugs indicates the necessity to check YB-1 levels in patients’ cancer specimens before applying CPT-containing combination strategies.

Another key issue awaiting further declaration is the structural basis for the interaction of YB-1 with TOPO1, but not TOPO2. According to the previous reports, TOPO1 is monomer while TOPO2 is homodimer in subunit structure in cells [53]. N-terminal and core domains of TOPO1 [54] and C-terminal domain of TOPO2 [55] are, respectively, considered important platforms for interaction with other proteins, and there is no sequence homology between the involved domains [53]. Despite there indeed several proteins could bind both proteins, most TOPO1-binding proteins could not bind with TOPO2. Moreover, TOPO1 and TOPO2 functionally are never acting in the replicative complex area at the same time, and it seems that monomer TOPO1 catalyzes a break in one strand of DNA duplex and involves in origin firing, while homodimer TOPO2 forms pre-replicative complex and generates a double-stranded gap in a DNA [53,56]. YB-1 has been reported to interact with the origin of single stranded DNA and its binding proteins for DNA regulation [57,58]. All of these provide structural and functional supports for the interaction of YB-1 with TOPO1, but not TOPO2. Furthermore, N-terminal or core domain of TOPO1 might be responsible for the specific binding, the identification of which would be helpful to improve our understanding of the physiological and pathological role of YB-1-TOPO1 interaction in cells.

## Conclusions

Taken together, our results reveal a new function for YB-1 and a novel mechanism of TOPO1 regulation: YB-1 induces cellular sensitivity to the TOPO1 inhibitor CPT and promotes the DNA relaxation potential of TOPO1 via a direct interaction between the two proteins. The interaction also acts as a cellular response to chemotherapy-induced oxidative-stress. Therefore, YB-1 functions as an essential biological response modifier of intra-nuclear TOPO1 and a novel regulator of CPTs efficacy on cancer cells. The current study could assist the development of better chemotherapy strategies to treat cancer and enable clinicians to determine which patients’ would benefit most from CPT-based treatment regimens.

## Abbreviations

YB-1: Y-box binding protein 1
TOPO1: DNA topoisomerase 1
TOPO2: DNA topoisomerase 2
CPT: Camptothecin
ADM: Adriamycin
VP-16: Etoposide
GST: Glutathione *S*-transferase
IPTG: Isopropyl-beta-D-thiogalactopyranoside
NAC: N-acetyl-L-cysteine
CSD: Cold shock domain
ROS: Reactive oxygen species
